# Genomic insights and emerging therapeutics in community-associated MRSA

**DOI:** 10.1097/MS9.0000000000004536

**Published:** 2025-12-09

**Authors:** Syeda Tayyaba, Umair Ali, Muhammad Ijaz Khan, Iman Osman Abufatima

**Affiliations:** a Department of Medicine, Azad Jammu and Kashmir Medical College, Muzaffrabad, Pakistan; b Department of Pharmacy, University of Swabi, Swabi, Pakistan; c Faculty of Medicine, University of Medical Sciences and Technology, Khartoum, Sudan

*To the Editor,*

Community-associated methicillin-resistant *Staphylococcus aureus* (CA-MRSA) remains a significant public health challenge due to its genetic adaptability and community spread. A recent genomic study by Smith *et al* analyzed pediatric CA-MRSA isolates in Spain, identifying high lineage diversity and the dominance of ST8-IVc clones that harbor multiple plasmid replicons^[1]^. Their work underscores the importance of genomic surveillance in delineating molecular epidemiology and resistance determinants in community settings. Similar shifts have been observed worldwide, with renewed increases in MRSA bloodstream infections and the emergence of evolving clonal complexes after a decade of apparent decline^[2]^. The persistence of CA-MRSA is driven by mobile genetic elements, such as SCCmec IV and plasmid-borne resistance cassettes, which enhance transmissibility and antibiotic tolerance^[3]^. Advances in 2025 have also highlighted emerging counter-strategies, including the discovery of artificial intelligence (AI)-designed small molecules capable of reversing β-lactam resistance and inhibiting MRSA biofilms in preclinical models^[4]^. Parallel efforts in phage therapy, antimicrobial peptides, and nanoparticle-based delivery systems have shown synergistic activity against multidrug-resistant *S. aureus* strains^[5]^. Environmental and behavioral contributors, particularly the role of shared community facilities and fomites, remain critical in sustaining CA-MRSA transmission, as emphasized by environmental genomic tracing studies^[6]^.

Furthermore, long-term resistance surveillance reports reveal co-resistance patterns and clonal evolution across northern Europe, reinforcing the need for harmonized genomic monitoring frameworks^[7]^. These findings collectively indicate that the future of MRSA control will depend on an integrated framework that combines genomic surveillance, rapid diagnostics, and novel therapeutics, rather than relying on conventional decolonization or antibiotic rotation. In this context, incorporating whole-genome sequencing into routine pediatric MRSA surveillance and expanding research on resistance-modifying therapeutics could substantially strengthen global infection-control strategies. This manuscript complies with the TITAN Guidelines, 2025, declaring no use of AI^[8]^.

## Data Availability

All data supporting the findings of this study are available within the article. The study is based on previously published literature; no new data were generated or analyzed.
